# ^1^H NMR Metabonomics Indicates Continued Metabolic Changes and Sexual Dimorphism Post-Parasite Clearance in Self-Limiting Murine Malaria Model

**DOI:** 10.1371/journal.pone.0066954

**Published:** 2013-06-24

**Authors:** Arjun Sengupta, Soumita Ghosh, Shobhona Sharma, Haripalsingh M. Sonawat

**Affiliations:** 1 Department of Chemical Sciences, Tata Institute of Fundamental Research, Mumbai, India; 2 Department of Biological Sciences, Tata Institute of Fundamental Research, Mumbai, India; National Institutes of Health, United States of America

## Abstract

Malaria, a mosquito-borne disease caused by *Plasmodium* spp. is considered to be a global threat, specifically for the developing countries. In human subjects considerable information exists regarding post-malarial physiology. However, most murine malarial models are lethal, and most studies deal with acute phases occurring as disease progresses. Much less is known regarding physiological status post-parasite clearance. We have assessed the physiological changes at the organ levels using ^1^H NMR based metabonomics in a non lethal self-clearing murine malarial model of *P. chabaudi* parasites and Balb/C, far beyond the parasite clearance point. The results showed distinct metabolic states between uninfected and infected mice at the peak parasitemia, as well as three weeks post-parasite clearance. Our data also suggests that the response at the peak infection as well as recovery exhibited distinct sexual dimorphism. Specifically, we observed accumulation of acetylcholine in the brain metabolic profile of both the sexes. This might have important implication in understanding the pathophysiology of the post malarial neurological syndromes. In addition, the female liver showed high levels of glucose, dimethylglycine, methylacetoacetate and histidine after three weeks post-parasite clearance, while the males showed accumulation of branched chain amino acids, lysine, glutamine and bile acids.

## Introduction

Malaria is caused by the five species of *Plasmodium*, namely, *P. falciparum, P. vivax, P. ovale, P. malariae* and *P. knowlesi* [1]. The infection may or may not affect the central nervous system. However, several studies reported post-malarial syndromes including neurological complications [2]–[6] and death is reported due to both cerebral and non-cerebral malaria [7]. Thus recovery from the malarial parasite infection may alter the host physiology significantly. Therefore, it is important to understand the organ level physiological events which are known to control and/or are affected during the recovery from the disease.

^1^H Nuclear Magnetic Resonance (NMR) spectroscopy based metabonomics offers a convenient way to understand the system level physiological changes by monitoring the composition of metabolites present within a particular biological compartment, such as an organ and/or biofluid. Metabonomics, which aims at understanding the global, dynamic metabolic response of living systems to biological stimuli or genetic manipulation [8], has been used to investigate diabetes [9], tuberculosis [10], cancer [11], and schistosomiasis [12]. The focus of these studies has been the identification of biomarker for the disease and/or to understand the pathway level perturbation during the disease process. Malarial infection, primarily, is not a metabolic syndrome; however, metabolic disorders arise as associated complications. For example, the human malarial parasite *P. falciparum* is known to sequester the blood vessel of several organ systems during the acute stage of the disease, affecting the microvasculature of the tissue systems such as liver, lungs, kidney, heart, eyes, intestine and adipose tissue [13]–[15]. During the late stage, a range of complications such as liver damage, cerebral malaria, renal damage, severe anemia, hypoglycemia and acidosis appear, probably through the severe inflammatory immune responses that often lead to death [16]–[18]. The physiological mechanism of transition to these conditions remains unclear. However, most of them are associated with alterations in the metabolism of the host. Therefore, it could be hypothesized that monitoring these alterations may provide insights into the pathophysiology of disease progression. A comparison of glucose utilization by the infected and uninfected RBCs was achieved using ^13^C NMR spectroscopy [19]. In addition, NMR based metabonomics was also employed to decipher the host metabolic response during early and late stage of malaria with sex based differences [20] and to understand the process of pleural effusion during the cerebral malaria [21] in lethal mouse model. The ^1^H NMR profile of urinary samples of *P. vivax* infected patients were also explored to discover candidate biomarkers for the disease [22]. However, the process of recovery from the infection remains unexplored till date.

In this paper, we report the metabolic changes during the peak infection stage and post-parasite clearance compared to healthy animals in a non-lethal murine model of the disease. Specifically, using Balb/C mice infected with *P. chabaudi.* Our data suggests that the recovered mice exhibit specific physiological differences from that of the healthy control mice. Moreover, the mode of physiological changes during peak infection as well as recovery is distinctly different in male and female mice.

## Materials and Methods

### Animal Experiments

The study was approved by the Animal Ethics Committee of the Tata Institute of Fundamental Research (IEAC approval no. TIFR/IEAC/2012-6, dt. 18-2-2012). The animals used in these experiments were treated as per the guidelines of the Animal Ethics Committee.

#### Rational for the experiment design

The experiments primarily focus on comparing three groups, uninfected controls animals, animals at the peak infection stage and three weeks post-parasite clearance (recovered state). There were two time points for the dissection of animals, viz. at the peak infection stage and recovered state. The number of control animals dissected were equally distributed among these two time points. Further care was taken to compensate for the age related changes owing to the time difference between these two points. Therefore, we made sure that number of animals remain same in each group under observation. Hence a parallel experiment was necessary on uninfected control mice where they were followed for age related temporal changes. The details of the experiments are described as under.

#### Collection of organs and serum

Twelve male and 12 female Balb/C mice (age 6–8 weeks) were used for the experiment. These mice were housed 4 mice per cage (males and females separately) and had free access to water and standard food pellets. No further acclimatization period was required because the mice caged together came from the same litter. The mice were maintained at 22±2°C under 12 hour day-night cycle. Eight mice from each sex were injected with ∼10^6^ *P. chabaudi* infected RBCs intra-peritoneally. The parasitemia for all the mice was followed every alternate day by counting 400–500 RBCs of the Giemsa stained blood smear with blood obtained from tail bleeding. At the peak stage of infection (∼25%–30% parasitemia), 4 infected female and 4 infected male mice were sacrificed along with 2 healthy control mice from each sex. The peak stage of infection was found to be the day8 for both the sexes. Therefore, this served as the first dissection point for the mice. Parasitemia was further followed for the rest of the infected mice and the time point (day16–20 p.i. (post infection), day16 for all the female mice, day18 for 1 male mouse and day20 for rest of the male mice) were recorded when the parasites were completely cleared off from the blood. A further three weeks of recovery time was allowed for the animals, after which they were sacrificed along with the control mice. Therefore, 4 female mice were sacrificed at day37, one male mice was sacrificed at day39 and three male mice were sacrificed at day41. Therefore, the difference between the two dissection point was 29 days for all the female mice, 31 days for one male mouse and 33 days for three male mice. Thus between two dissection points, there is an age difference of ∼1 month. In the parallel experiment, to compensate for the age related changes, 8 male and 8 female uninfected Balb/C mice (grown in the same laboratory condition and littermates) were followed over a time period of four weeks. At day zero 4 male and 4 female mice were sacrificed and the rest of the mice were sacrificed after four weeks. This therefore helped in aligning the sampling event in the actual experiment and therefore took care of the age related changes in the actual experiment.

To collect the serum, the animals were anesthetized using ether and the blood was collected using retro-orbital bleeding of the animal using non-heparinized capillary tubes. The blood samples were collected directly into microtubes from the capillary. No anticoagulant was used during this procedure. Approximately 700 µl blood could be drawn from each animal. The blood samples were centrifuged at 13,000 g for 10 minutes and the supernatant was collected. This was stored at −80°C for further processing.

Immediately after the collection of blood, the animals were sacrificed by cervical dislocation and the liver and brain were dissected out, snap-frozen in liquid nitrogen, carried to the working laboratory and stored at −80°C till further use for sample preparation for NMR spectroscopy.

### Preparation of Methanol/Chloroform/Water Extracts of Organ and Serum Samples

The organ samples were prepared by a slight modification of the method suggested by Beckonert et. al. [23]. Briefly, intact frozen tissue was weighed; 4 ml g^−1^ methanol was added to it followed by 0.85 ml g^−1^ water. The suspension was vortexed and 2 ml g^−1^ chloroform and 2 ml g^−1^ of water was added. This tissue suspension was homogenized using tissue homogenizer (Wheaton, USA), left for 15 minutes at 4°C and centrifuged at 1000 g. The two layers (methanol/water and chloroform) were collected separately. The methanol/water fraction was dried off using vacuum concentrator and reconstituted in 600 µl D_2_O (served the purpose of field frequency lock during NMR experiments) and 100 µl D_2_O containing 0.03% (w/v) TSP (3-(trimethylsilyl)-2,2′,3,3′-tetradeuteropropionic acid, used as a stock TSP solution). The samples were immediately used for NMR experiments.

To extract the serum samples, 200 µl of methanol, 300 µl of chloroform and 450 µl of water was added to 200 µl of crude serum. This mixture was vortexed and 600 µl of methanol/water aliquot was obtained from each of the samples. These fractions were dried by vacuum concentrator and reconstituted in 600 µl D_2_O and 100 µl D_2_O containing 0.03% TSP. The samples were immediately used for NMR experiments.

### ^1^H NMR Spectroscopy of Organs and Serum

700 µl of extract of organs and serum were taken in 5 mm NMR tubes (Norrell Inc.) and loaded onto the BACS automation system installed with a Bruker AVANCE 700 MHz NMR spectrometer, fitted with a broad band inverse probe (Bruker Biospin, Karlsruhe), the temperature of the probe was maintained at 298 K during the experiments. ^1^H NMR spectra were acquired (including the shimming and tuning/matching of the probe) and processed using the ICON NMR automation software. The pulse program used was the first trace of the NOESY (Nuclear Oherhauser Effect SpectroscopY) experiment which takes the form RD-90°-τ-90°-TM-90°-ACQ, where RD is the relaxation delay of 4 seconds, τ represents a very short delay (3 µs) and TM is the mixing time (150 µs). The remnant water signal was saturated for each sample by continuously exciting the water resonance during the relaxation delay and the mixing time. Spectral window of 20 ppm was used for the acquisition with 64 k data points resulting in an acquisition time of 2.28 s for each FID. A total of 64 such scans were co-added resulting in an experiment time of ∼3.5 minutes for each spectrum. For processing, an additional line broadening factor of 0.3 Hz was applied and the FIDs were Fourier transformed. The phase and baseline corrections were achieved by an automation program kindly made available by Dr. Eberhard Humpfer of Bruker Biospin.

In order to identify and assign the metabolites, 2 dimensional NMR experiments such as COSY (Correlation SpectroscopY), TOCSY (Total Correlation SpectroscopY) and HSQC (Heteronuclear Single Quantum Coherence) were performed. COSY and TOCSY were recorded with 2048 and 512 data points in the direct and indirect dimensions, respectively. Each increment in the indirect dimension was consisting of 32 FIDs (Free Induction Decay). In both the dimensions, 12 ppm of spectral window was used. For each spectrum 2 seconds of relaxation delay was employed, during which the water resonance was saturated by continuous irradiation. For processing sine square window function was used in both the dimension with a line broadening of 0.3 Hz and a Gaussian multiplication of 0.1 Hz in the indirect dimension, following which, the FIDs were Fourier transformed. The TOCSY spectra were phase corrected manually, whereas both COSY and TOCSY spectra were baseline corrected automatically using TOPSPIN 2.1 (Bruker Biospin, Switzerland). For acquiring the HSQC spectra, 2048 and 128 data points were acquired in the direct and indirect dimensions, respectively. Each increment in the indirect dimension was consisting of 128 FIDs in the direct dimension. The spectral windows in the direct and indirect dimension were 16 and 220 ppm respectively. A relaxation delay of 2 seconds was employed. For processing, sine square window function was used in both the dimensions with line broadening of 1 and 0.3 Hz along the direct and indirect dimensions, respectively and a Gaussian multiplication of 0.1 Hz along the indirect dimension. These spectra were phase corrected manually and automatically baseline corrected using Topspin 2.1 (Bruker Biospin, Switzerland).

### Statistical Analyses of the Data

#### Multivariate data analysis

Multivariate data analyses were carried out using AMIX 3.8.4 (Bruker Biospin, Switzerland) and SIMCA-P+12.0 (Umetrics, Sweden). Initially, the spectra were imported from Topspin 2.1 to AMIX and all the spectra were binned into 0.04 ppm frequency bin. The region consisting of the water resonance (4.5–5.1 ppm) was excluded from this procedure. Each bin from each spectrum was integrated, normalized to the total intensity of the spectrum, mean centered and Pareto scaled. This gives rise to the working data matrix, which was imported into SIMCA-P+12.0 for further statistical analysis.

The data matrix was subjected to Principal Component Analysis (PCA). In addition to the pattern analysis, this also served as the initial check on the data so as to find out any hidden trend(s) and outliers. PCA is an unsupervised method with no previously assigned class entity to the sample set. This is, primarily, a dimension reduction technique, where the multidimensional and highly correlated data matrix is reduced to small number (2/3) of orthogonal components known as the principal components (PCs). The fraction of the total variation in the data explained by a PC can be deduced from the R^2^X associated to that PC. Overall variation explained by the model is the sum of variation explained by all the PCs and is interpreted by the cumulative R^2^X(cum). Each PC is a linear combination of the original variable, therefore, each sample can be projected onto the newer, 2/3 dimensional space of the PCs and a pattern can be identified; this is visualized in terms of the scores plot. In addition, the contribution of individual variables on a certain PC can be identified using the loadings plot. The loadings plot, hence, serves as the identifier of the spectral variables/bins which are significant in regards to the trend observed in the scores plot.

In the present study, 12 separate PCA models were created, 2 each for a single biological compartment (liver/brain/serum) comprising of the control vs peak infection and the control vs recovered (three weeks post-parasite clearance) classes. This procedure resulted 6 models from each sex *viz.* 2 models from live profile of control-peak infection and control-recovered, similarly for brain and serum. The scores and loadings were analyzed from the individual models. The bins which were identified by this procedure to be significant for certain class separation were further analyzed for the metabolite entities using the 2- dimensional NMR experiments and database search (Human Metabolome database- www.hmdb.ca, Madison Metabolomics Consortium database - www.mmcd.nmrfam.wisc.edu ). The relevant metabolites peaks in the original NMR profiles were further integrated using AMIX and individual peaks compared by univariate analysis to look for statistically significant difference across the classes.

#### Peak integration and univariate data analysis

After obtaining the bins contributing significantly towards the clustering of certain classes, they were analyzed by AMIX and the peak(s) corresponding to the bin(s) was/were integrated and normalized to the total spectral intensity. These peak integrals were then compared across the two concerned classes by Student’s t-test using MS Excel. In order to check whether the bins are significantly affected by the infection or they are varying because of the temporal change of ∼4 weeks between the time periods, the same peaks were integrated in the same manner from the second set of experiments where uninfected mice were followed temporally over four weeks and they were compared. Only the data corresponding to bins that were not found to be significantly changing across the 4 weeks window in uninfected animals is presented here.

## Results

**Figure 1:**
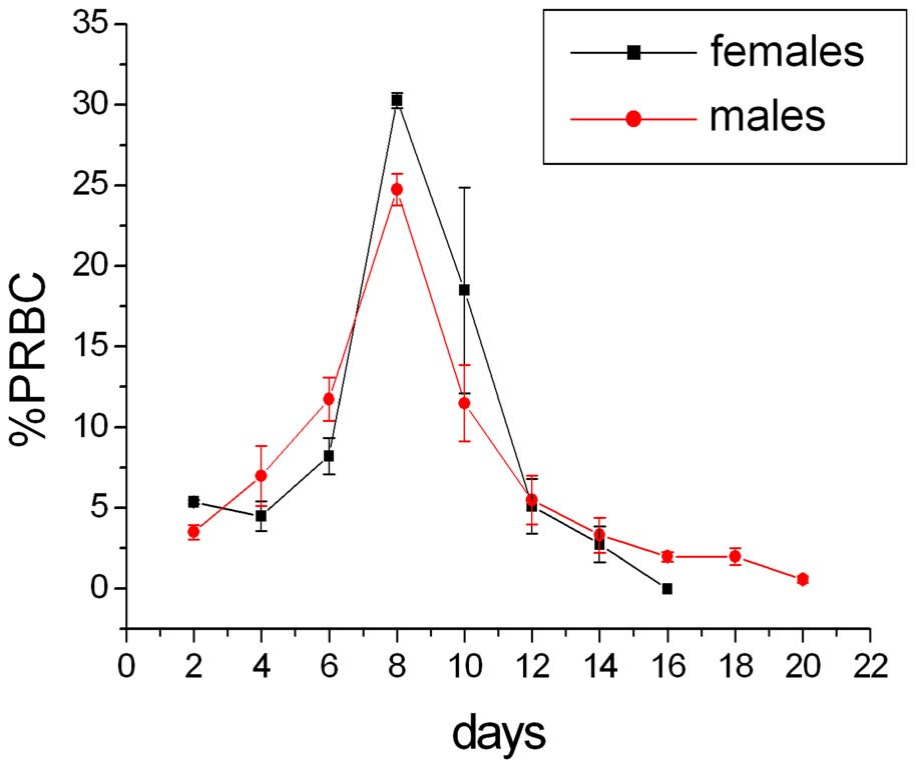
The parasitemia kinetics of *P. chabaudi* infected Balb/C mice. The parasitemia was calculated from RBCs counted on Giemsa stained blood smear. 400–500 RBCs were counted in each case.

**Figure 2:**
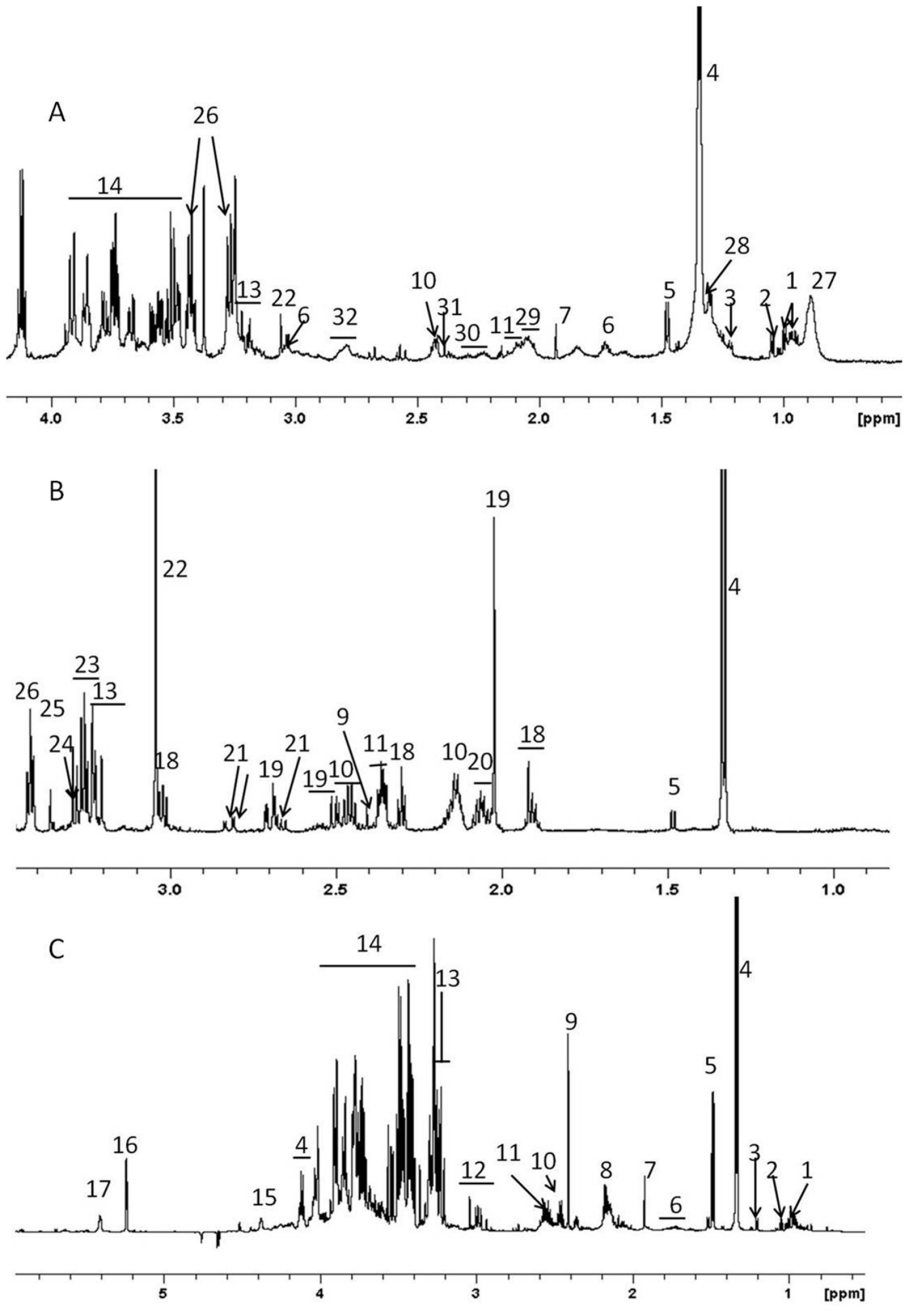
Representative 700 MHz ^1^H NMR spectra of hydrophilic fraction of methanol/water/chloroform extract. (A) serum, (B) brain and (C) liver from healthy uninfected mouse. Only aliphatic region of the spectra is shown. 1- Isoleucine/Leucine, 2- valine, 3- 3-hydroxybutyrate, 4- lactate, 5- alanine, 6- lysine, 7- acetate, 8- glutamate/glutamine, 9- succinate, 10- glutamine, 11- glutamate, 12- aspartate+lysine, 13- N^+^(CH_3_)_3_ head groups from GPC, PC and choline, 14- carbohydrates, polyols and amino acid ά-^1^H, 15- N-methylnicotinamide, 16- anomeric ^1^H of ά-glucose, 17- glycogen, 18- γ-aminobutyric acid, 19- N-acetylaspartate, 20- glutamate+N-acetylneuraminate, 21- aspartate, 22- creatine, 23- taurine+myo-inositol, 24- myo-inositol, 25- scyllo-inositol, 26- taurine, 27- lipid (LDL/VLDL- CH3(CH2)n),28- fucose, 29- lipids (CH2-C = C), 30- lipids (CH2-C = O), 31- oxaloacetate/pyruvate, 32- = C-CH2-C = .

The parasitemia of the infected animals (8 males and 8 females) were followed every alternative day (Figure 1). Both the sexes showed peak parasitemia around day8 post-infection which was 30.25±0.48% for the females and 24.75±0.97% for the males. The parasite clearance was observed at day16 for the females and day18–20 for the males (Figure 1). Four animals from each sex were allowed a further recovery for three weeks post clearance of the parasite and they were then sacrificed and serum, liver and brain were collected. NMR spectra were recorded after extracting the serum and organs using methanol/chloroform and water and multivariate analyses were carried out. Figure 2 shows the representative assigned ^1^H NMR spectral profile from the liver, brain and serum extracts from a healthy female uninfected mouse.

### Metabolic Alterations at Peak Infection and Three Weeks Post-parasite Clearance Stage

Initially, PCA was performed on all the samples belonging to a single biological compartment to find trends and outliers, if any. Certain trends were indeed observed in the scores of the PCA models of individual organ/biofluid of either male or female mice, comprising of three different stages of the disease. For example, in both the sexes, liver profiles were distinct across the three stages. Female serum profiles were also distinct across the three stages. However, no distinctive feature was observed in the brain profile across the three stages of either sex (Figure S1). Instead, the male brain and serum profile showed dominating temporal behavior. This is evident since the samples profiled at two different time points are segregated by the PC1 (S1E and F).

**Figure 3:**
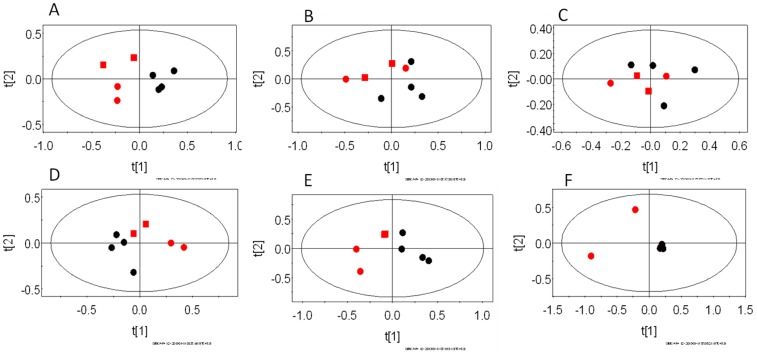
The PCA scores plots of ^1^H NMR spectra of Balb/C mice infected with *P. chabaudi* illustrating the segregation of uninfected control animals from animals at the peak infection stage. The scores plots generated from PC1 and PC2 are illustrated here. The plots represent pair-wise models of different biological compartments. A–C females and D–F males. A and D - liver, B and E- serum, C and F–brain. In each plot, red symbols are uninfected control mice and black dots represent animals at peak infection stage. Among the red symbols, the squares represent the control mice sacrificed along with the peak infection animals and the circles represent the animals sacrificed with the three weeks post-parasite clearance stage animals.

**Figure 4:**
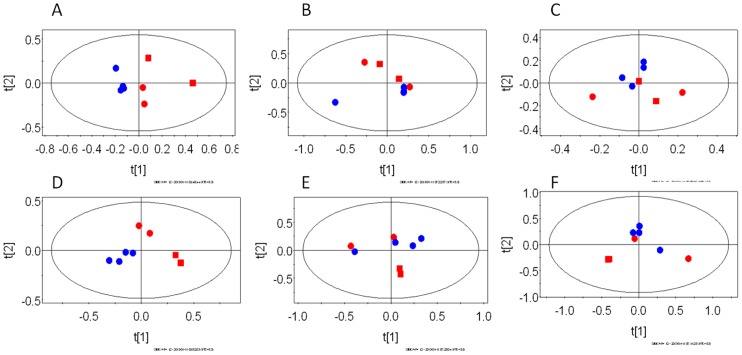
The PCA scores plots of ^1^H NMR spectra of Balb/C mice infected with *P. chabaudi* showing the segregation of uninfected control from infected mice three weeks post-parasite clearance. The recovered time point was taken as the three week post parasite clearance. The plots represent pair-wise models of different biological compartments. A–C represent females and D–F males. A and D- liver, B and E – serum, C and F –brain. In each plot, the red dots are uninfected control and the blue dots represent the animals at three weeks post-parasite clearance. Among the red symbols, the squares represent the control mice sacrificed along with the peak infection animals and the circles represent the animals sacrificed with the three weeks post-parasite clearance animals.

Further, PCA models were constructed for pair-wise analysis of the classes and identification of spectral bins responsible for class discrimination. A total of 12 such models were created viz. for each biological compartment (liver/brain/serum), uninfected control animals were compared with animals at peak infection stage and three weeks post-parasite clearance. The statistical parameters (R^2^X) for individual models are given in the table 1. Figures 3 and 4 depict the PCA scores plots showing the segregation of individual biological compartments of the control animals from those at peak infection stage and post-parasite clearance stage, respectively.

**Table 1:** The explained variations of the PCA models generated from the liver, brain and serum of the male and female mice.

PCA Model	Sex	Liver	Brain	Serum
		R^2^X	R^2^X	R^2^X
Uninfected & peak infection	Male	0.49 (PC1)	–	0.35 (PC1)
	Female	0.45 (PC1)	–	0.31 (PC2)
Uninfected & 3 weeks post-parasite clearance	Male	0.50 (PC1)	0.30(PC2)	0.09 (PC4)
	Female	0.45 (PC1)	0.28 (PC2)	0.26 (PC2)

Twelve models were created. For each sex, liver, brain and serum NMR profiles at the peak infection stage and three weeks post parasite clearance were compared with the uninfected control animals. The Principal Components showing the relevant variation are listed within brackets. No significant infection related variation was observed in the brain while comparing uninfected controls and peak infection animals in both the sexes. Hence, no data is given.

Several significant bins varying across the relevant classes could be identified from the loadings plots of the PCA. Metabolites corresponding to these bins were assigned using the 2-dimensional NMR spectroscopic techniques such as COSY, TOCSY, JRES and HSQC performed on selected samples. In certain bins more than one metabolite could be identified. All such metabolites were grouped according to the organ/biofluid and sex. This resulted in six sets of metabolites (three biological compartments for each sex). Levels of each metabolite in a particular set were compared by univariate analysis (Student’s t- test) in two parallel subsets – uninfected control animals vs. peak infection stage and uninfected control animals and post-parasite clearance stage. Significantly altered metabolites are reported in Table 2. The relative levels of alteration in the pair-wise test for individual metabolites are provided in Figures S2 and S3. This procedure revealed that certain metabolites which did not show up in the PCA models as highly significant are indeed significantly varying across the two relevant classes. Thus creatine and alanine were found to be significantly altered in the serum of female and male animals, respectively, at the post-parasite clearance stage (Table 2). Combining the multivariate and univariate data, several variations were observed in the levels of metabolites which are delineated in the sub-sections that follow. The CheBI ID of the all the altered metabolites are provided in Table S1.

**Table 2:** Metabolites significantly contributing to the separation of liver, brain and serum^ 1^H NMR profiles of uninfected control Balb/C mice from mice infected with *P. chabaudi* at peak infection or three weeks post-clearance of the parasite.

Peak Infection vs uninfected control				Three weeks post-parasite clearance vs. Uninfected controls			
↑	p	↓	p	↑	p	↓	p
Liver							
Male							
Glucose*	−0.15	Lactate*	0.3	Glutamine^#^	−0.12	Glucose*	0.29
DMG*	−0.21	PCH*	0.13	L-DOPA*	−0.08		
				BA*	−0.11		
				Valine*	−0.11		
				Isoleucine*	−0.11		
				Leucine*	−0.07		
				Lysine*	−0.06		
				u/c*	−0.06		
				2-HI*	−0.09		
Females							
Glutamine^#^	0.20	GDA*	−0.20	Glucose^#^	−0.15	–	–
Glutamate^#^	0.16	Ascorbate^#^	−0.19	MAA*	−0.17		
L-DOPA^#^	0.12	MAA^#^	−0.15	DMG*	−0.17		
Valine^#^	0.11	DMG^#^	−0.15				
Isoleucine^#^	0.11	u/c^#^	−0.15				
Leucine^#^	0.09	Histidine^#^	−0.16				
Lysine^#^	0.10	Glucose^#^	−0.13				
BA^#^	0.10						
2-HI^#^	0.10						
NAG^#^	0.09						
Betaine^#^	0.11						
Brain							
Male							
–	–	–	–	ACH^#^	0.07	–	–
Female							
OAA*	0.16	–	–	PCH*	0.31	u/c*	−0.15
				ACH*	0.31	Methionine^#^	−0.04
Serum							
Male							
Glycerol*	0.07	Lipid*	−0.10	–	–	Alanine*	−0.04
		Alanine*	−0.08				
		NAG*	−0.04				
Female							
Lactate*	0.20	Glucose*	−0.14	–	–	Creatine*	0.05

The loading weights (p) of the original variable of the PCA scores (for the relevant PC, see result) are mentioned for each metabolite. The difference across the two classes were further subjected to univariate analysis (^*^p∼/<0.05, ^#^P<0.005). The variations of the metabolites during 4 weeks of experiment were compared with the uninfected animals in a parallel experiment. The metabolites that did not exhibit age related changes in levels are mentioned in this table. In case of multiple NMR peaks contributing only the most significant loadings value is provided.

Keys- DMG: Dimethylglycine, PCH: Phosphorylcholine, L-DOPA: L-3,4-dihydroxyphenylalanine, BA: Bile acids, 2-HI: 2-hydroxyisovaleric acid, NAG: N-acetylglutamate, GDA: Guanidoacetate, MAA: Methylacetoacetate, ACH: Acetylcholine, OAA: Oxaloacetate, u/c: Unconfirmed.

### Liver Metabolic Profile at Peak Infection and Post-parasite Clearance Stage Compared to Uninfected Control Mice of Same Sex

The liver profile of the males and females were found to be distinct during peak infection stage with respect to same sex control animals (Figure 3A and 3D for females and males, respectively). Both of the sexes showed distinctive features along the 1^st^ PC (Figures 3A and 3D). Some temporal behavior of the liver profile was also noted in these cases. For example, in the females, this behavior was captured in PC2. In males also, the control mice that were sacrificed together with peak infection stage were found to be closely spaced in the scores plot (Figure 3D) with the peak infection stage animals. The male mice showed elevated levels of glucose, DMG and decreased lactate and phosphocholine in the liver during the peak infection stage compared to same sex uninfected control mice (Table 2).

In the female mice, glutamine, glutamate, L-DOPA, the branched chain amino acids, lysine, bile acids, 2-hydroxyisovalerate, NAG and betaine were elevated along with decrease in guanidoacetate, ascorbate, methylacetoacetate, DMG, histidine and glucose (Table 2).

The liver profiles remained distinct in both the sexes three weeks post-clearance of the parasite (Figure 4A and 4D, for females and males, respectively). In this case also, the distinctive features were observed along the 1^st^ PC. The temporal groupings were also found in this case in both the sexes, although the infection/recovery related changes remained the dominating factor. Levels of several metabolites were also altered significantly. For example, males showed elevated levels of glutamine, L-DOPA, bile acids, branched chain amino acids, lysine and 2-hydroxyisovalerate along with decreased glucose while the females showed elevated levels of glucose, methylacetoacetate, DMG (Table 2).

### Brain Metabolic Profile at Peak Infection and Recovered Stage Compared to Uninfected Control Mice of Same Sex

The brain profile of the females at the peak infection stage remained largely indistinguishable from the uninfected controls (Figure 3C). For males also, the difference was not significant for the brain profile (Figure 3F). Instead the males showed a temporal variation in their brain profile. Thus, the two uninfected control animals that were sampled with the three weeks post parasite clearance animals were grouped separately along the 1^st^ PC. As expected, no metabolites were also significantly altered except a slightly higher level of oxaloacetate in female mice during the peak infection stage (Table 2). However, certain variation in the brain profile three weeks post-clearance were indeed observed when compared to the uninfected control animals (Figure 4C and 4F, for females and males, respectively, along 2^nd^ PC in both the cases). In both the cases, very little temporal variation was observed. When the individual metabolites were compared, males showed higher level of acetylcholine (Table 2). Females showed high level of phosphocholine and acetylcholine, along with lower level of methionine, compared to control animals (Table 2).

### Serum Metabolic Profile at Peak Infection and Recovered Stage Compared to Uninfected Control Mice of Same Sex

The serum profiles of the animals also showed some distinction during the peak infection stage with respect to the same sex control animals (Figure 3B and 3E, for females and males, respectively). The PCA scores plot for the females showed less distinction than that of the males. In the later case, the distinction was observed along the 1^st^ PC. However, the univariate analysis captured several significant differences in certain metabolite levels. For example, the serum level of glycerol was high along with lower levels of lipid, alanine and NAG in the male mice, while female mice showed high level of lactate and low level of glucose during the peak infection stage (Table 2). Post-parasite clearance, the distinction with respect to the control mice were more evident in the females (that was captured on the 2^nd^ PC) than that of the males. Males showed very weak distinction (could be captured to some extent on the 4^th^ PC, data not shown). Univariate analysis of the relevant loadings from the PCA revealed low level of creatine in the female mice and slightly low level of alanine in the serum of the male mice (Table 2).

## Discussion

We have studied the alterations in the metabolome of liver, brain and serum during infection and recovery of Balb/C mice infected with *Plasmodium chabaudi*. This model of malaria has been of particular interest because of its non-lethality and resemblance to non-lethal human malarial infection caused by *P. falciparum* and *P. vivax* [24]. In addition to the similar survival rate, this mouse strain shares significant hematological similarities to *P. falciparum* [25], [26]. The similarity in the immune mechanisms with the human infection led to extensive research using this strain of the parasite [24]. Non-lethal murine malaria models are not investigated much during the process of recovery, although, this could be important due to several reasons. For example, relapse of *P. vivax* malaria due to the existence of hypnozoites in the host system is a known phenomenon [27]. Post malarial neurological syndrome is yet another clinical concern among researchers [4]–[6]. Understanding the host physiology during various stages of infection is a pre-requisite to address these issues. As an organ that regulates the metabolism, liver metabolic profile offers insight into the changes brought in by external stressors and also the regulation undertaken by the host to maintain homeostasis. The homeostasis or its perturbation can be observed readily through serum metabolic profile. In addition, brain metabolite profiles were also investigated which might provide information on the pathophysiology of the post-malarial neurological syndrome (PMNS).

Earlier studies using other malarial models documented infection-induced changes in the host metabolism during disease progression [20]–[22], [28]. This study of the self-limiting model, also reports similar changes at peak infection point, when the metabolic profile of liver and serum are compared with that of uninfected control mice.

As reported before [20], during the peak infection stage, the metabolic profiles were altered significantly (Figure 3A–F). However, certain features across the two sexes were captured in the organ level changes in the metabolic profile. For example, although the liver profiles were significantly different (Figure 3A and 3D) in both the sexes, female mice showed less perturbation in the serum profile than that of the males (Figure 3B and 3E). This corroborates with previous results of early stage lethal malarial model that reported successful maintenance of blood homeostasis by the female mice compared to the males [20].

Three weeks post-parasite clearance, the liver profile remained clearly distinct from that of the control animals in either sex. Moreover, in both the sexes, the brain showed variation in the global profile. The serum profile remained distinct to some extent in the females while the males seem to bring back the homeostatic condition in the serum metabolic profile.

**Figure 5:**
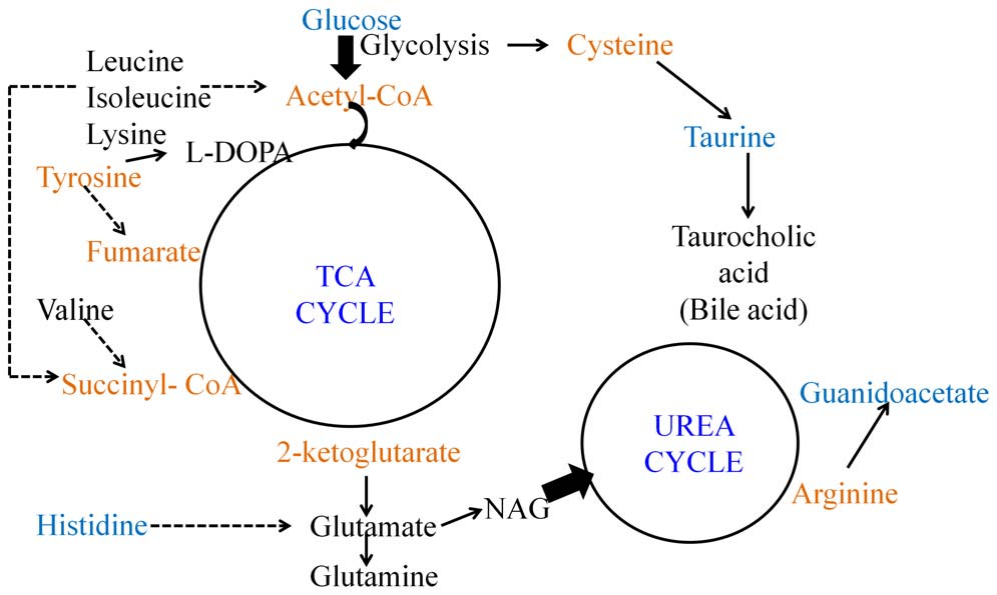
The probable metabolic network affected during the peak infection stage in the female liver. Metabolites marked in black are elevated during the peak infection stage and those in blue are lowered at this stage. Metabolites in orange font did not show any significant variation and/or not detected in the ^1^H NMR spectra.

Both male and female liver profiles were altered significantly as compared to the uninfected control animals. This is evident from the PCA scores plots (Figure 3A and 3D). Levels of several metabolites were altered in the female liver profile significantly during the peak infection stage. Females showed elevation in the level of glutamine, glutamate, L-DOPA, branched chain amino acids, lysine, 2-hydroxyisovalerate, N-acetylglutamate and betaine. They also showed lower level of guanidoacetate, ascorbate, methylacetoacetate, DMG, histidine and glucose (Table 2). Together, this indicates a perturbation of an extensive metabolic network which includes the central carbon metabolism such as the TCA cycle and the pathways converging onto/branching away from this, such as the branched chain amino acid and lysine degradation pathways, cysteine biosynthetic pathway as a branch-out from glycolysis and further conversion onto taurine and bile acids, conversion of 2-oxoglutarate to glutamate and glutamine, histidine degradation and conversion of tyrosine to L-DOPA. A schematic of the probable perturbed liver metabolism is provided in Figure 5. Although the pathophysiological consequences of these observations remain to be investigated, since the central carbon metabolism supplies both energy and intermediates necessary for other pathways, it is logical to interpret that the female mice are sensitized towards the peak infection stage and could adjust their liver metabolism to largely keep the serum homeostasis unaltered. In addition, we observed a statistically significant increase in the N-acetylglutamate level of the liver and a decrease in guanidoacetate (Table 2, Figure 5). Together, these indicate alteration in the urea cycle. N-acetylglutamate is an allosteric activator of the urea cycle and guanidoacetate is a product of degradation pathway of arginine, which is a major metabolite of the cycle. This condition may lead to hyperammonemia. However, it is possible that the enhanced synthesis of glutamine nullifies this effect. Males, on the other hand, showed completely different response in the liver during the peak infection stage. For example, they showed accumulation of glucose and decrease in the level of lactate (table 2). This might mean enhancement of gluconeogenesis in the male liver.

The serum profile showed some alteration in the female mice. In terms of the global profile, this was observed in the PC2 and PC3 (data not shown). However, these mice showed significantly elevated levels of lactate and decreased glucose level in their serum (Table 2). This suggests increased glycolysis during the peak infection stage. This is a complication reported earlier in malaria, specifically during heavy parasite load [19]. The effect seen on the male mice serum profile is greater than that of female mice (Figure 3E). This may reflect that the males are relatively more affected towards the peak infection. However, it is interesting to note that the male mice did not show significant depletion of blood glucose and build-up of lactate. This may be a consequence of elevated gluconeogenesis in the male liver that accounts for the glucose homeostasis and utilization of built-up lactate. Instead, these mice had elevated glycerol and lowered level of lipids in their serum (Table 2). This suggests an altered lipid parameter in serum of male mice which is also reported during progression of malaria [28], [29]. The *Plasmodium* parasites need enhanced phospholipid synthesis for their growth and proliferation in the host system. For this purpose a CDP-choline dependent pathway imports the choline precursors from the host [30]. This might explain the associated changes in the lipid parameters in the serum. We show this behavior to be different across the sexes. While in the females, elevation of glycolytic pathway is more prominent, the males showed a change in the lipid parameters. The relation of host lipid metabolism and the progression of the disease in the males is more evident from the altered liver metabolome. The male mice showed significantly elevated dimethylglycine and decreased phosphocholine during the peak infection stage (Table 2). This suggests inhibition of the phosphocholine synthetic pathways in the male liver during the peak infection stage. Therefore, unlike the females, the major response in the male liver during the peak infection stage may be to shut off the feeding pathways to the parasites. In addition to this, the male mice also showed changes in terms of decreased lactate and elevated glucose concentration the liver, suggesting inhibited glycolysis and elevated gluconeogenesis. This suggests that elevated liver gluconeogenesis could replenish the blood glucose hence counter-acting the blood glucose depletion as a result of enhanced glycolysis.

While analyzing the recovery of the mice three weeks post-parasite clearance with respect to the control mice, our data suggested that physiology of the post-parasite clearance mice is different from that of the uninfected control mice (Table 1). The differences that we recorded in terms of the altered metabolite levels in liver, serum and brain are not age related since we have compared the data here with that of uninfected control animals of appropriate age. The modes of host response in the two sexes seem to be clearly distinct. The serum of females showed significantly decreased creatine, which indicates altered energy metabolism, while males showed decreased alanine (Table 2). Furthermore, females showed certain distinction in the three weeks post-clearance serum profile (Figure 4B), while males did not. Therefore, this might mean that the global homeostasis is brought back in the males. Further differential sexual response was recorded from the liver metabolite profiles. Female livers after recovery were distinct (Table 1) in terms of significantly elevated glucose, methylacetoacetate, dimethylglycine (Table 2). Since no gluconeogenetic or glycolytic intermediary metabolites were significantly altered, this might mean alterations in the glycogenolysis and glucose accumulation during recovery. However, males showed significant changes in levels of several metabolites. Among them, glutamine, L-DOPA, bile acids, branched chain amino acids, lysine, and 2-hydroxyisovalerate were elevated while glucose level was significantly decreased (Table 2). This list of metabolites shared striking commonality with those perturbed in the female liver during the peak infection stage. This suggests that the central carbon metabolism and the associated pathways (Figure 5, except the urea cycle component) remain perturbed in the male mice during the recovery process. Possibly, longer recovery period from the infection is needed for both the sexes. The male animals specifically, manipulate their liver metabolism extensively in order to keep the serum homeostasis.

**Figure 6:**
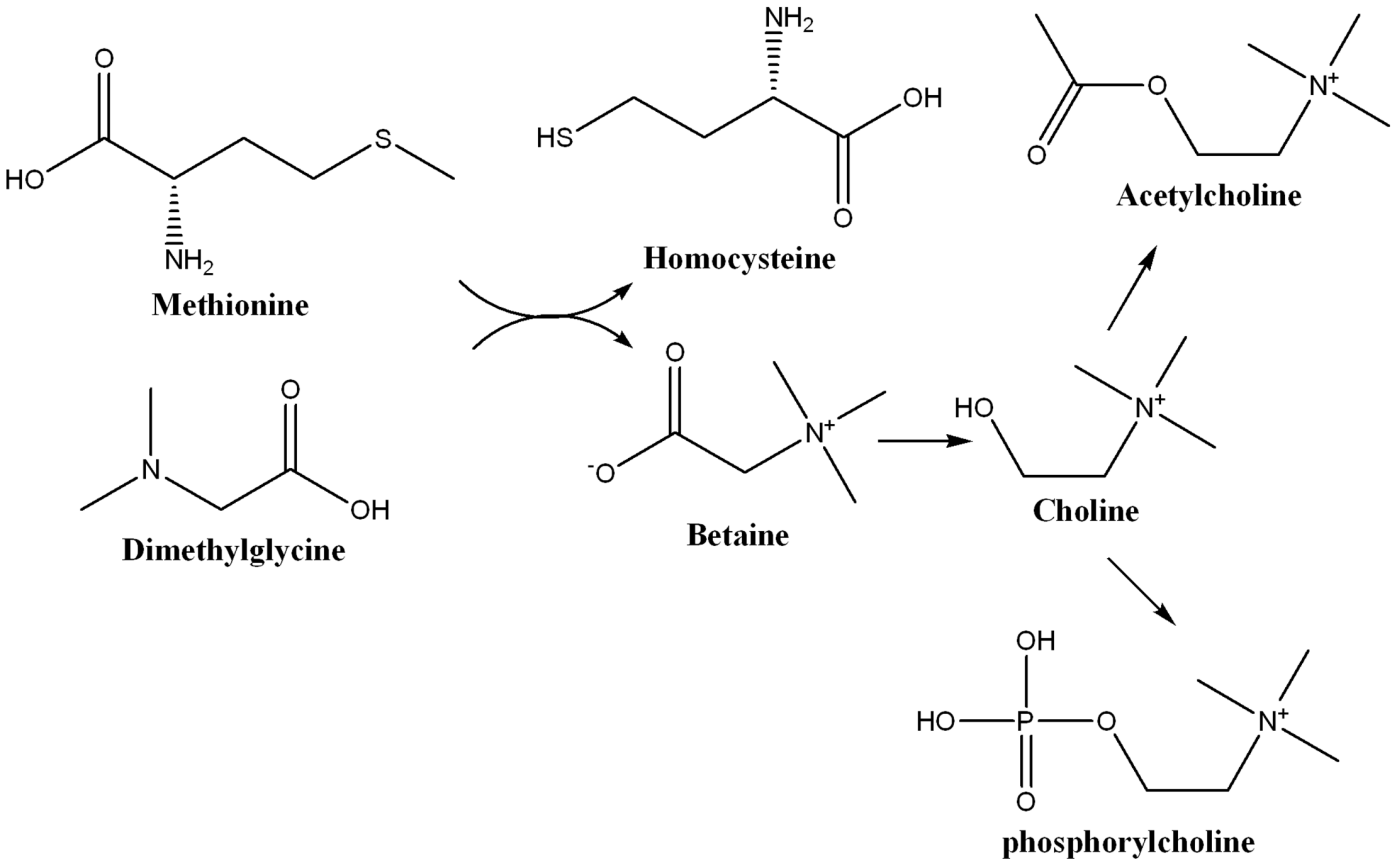
Schematic of acetylcholine biosynthesis in the central nervous system. Acetylcholine was found to be elevated in the both three weeks post-recovery male and female Balb/C mice infected with *Plasmodium chabaudi*. In addition, the female mice showed elevation in the level of phosphorylcholine and decrease in methionine.

We observed accumulation of acetylcholine in brain during the post-parasite clearance stage in both the sexes. The female brain, in addition, showed elevation of phosphocholine and decrease in the levels of methionine (Table 2). Therefore, our results suggest changes in the acetylcholine biosynthetic pathway (Figure 6) in these animals. Accumulation of acetylcholine, termed as the cholinergic burst, in the central nervous system is known to cause (cholinergic) crisis. This is a clinical condition arising out of inhibition of acetylcholinesterase hindering the hydrolysis of acetylcholine. In humans, prolonged cholinergic burst has been reported to exhibit several neurological symptoms that include tremors and respiratory dysfunction [31], epileptic dysfunction [32] and other neuropathology [33]. Neurological complications after recovery from malaria (PMNS) include symptoms such as generalized convulsion, acute confusional state, psychosis, tremor, cerebellar ataxia, generalized myoclonus etc [34]–[36]. Most of these symptoms could be attributed to alterations in acetylcholine level [37]–[40]. Therefore, significant alterations in the level of acetylcholine observed in this study may be connected to the pathophysiology of post malarial neurological complications.

### Conclusion

Recent studies have shown that metabolic phenotyping could be a potential key to understand the disease pathophysiology of malarial infection in animals and humans. However, the host physiological changes during various stages of infection, and more importantly during recovery, are not well understood. In this report we observed that the metabolic phenotypes in mice were significantly different from age and sex matched uninfected control mice, even when they were three weeks beyond the parasite clearance point. This is important in the context of the time span of recovery because three weeks is a significant duration in the mouse life-span. Moreover, the modes of transition to the post-recovery physiological status were also distinct across the two sexes in the mouse model of non-lethal malaria. The results described here may help improve our understanding of the post-malarial complications in the non-lethal forms of the disease.

## Supporting Information

Figure S1 **The PCA scores plots constructed from the NMR profiles of hydrophilic fractions of water/methanol/chloroform extracts.** Female (A–C) and male (D–F) liver (A/D), brain (B/E) and blood serum (C/F) are shown. All the plots are constructed from the 1st and 2nd PCs of the models. In each plot, red dots- uninfected control animals, black dots- peak infection stage animals and blue dots- three weeks post parasite clearance animals. The total explained variances by the 1st and 2nd PCs in each model are as follows- A- 0.72, B- 0.58, C- 0.59, D- 0.68, E- 0.83 and F- 0.57.
(TIF)

Figure S2 **Relative changes of levels of the significantly perturbed metabolites during the peak infection stage compared to the uninfected controls illustrated by boxplots.** A- male liver, B- female liver, C-male blood serum, D- female blood serum. The plots represents the comparison the NMR peak intensity normalized to total spectrum intensity (Y- axis) of the relevant metabolite peak at control (black) and peak infection stage (red) (X- axis).
(TIF)

Figure S3 **Relative changes of levels of the significantly perturbed metabolites during the three weeks post-parasite clearance stage compared to the uninfected controls illustrated by boxplots.** A- male liver, B- female liver, C- male brain, D- female brain. The plots represents the comparison of the NMR peak intensity normalized to total spectrum intensity (Y- axis) of the relevant metabolite peak at control (black) and three weeks post-parasite clearance stage (red) (X- axis).
(TIF)

Table S1 **CheBI IDs of the relevant metabolites reported.**
(DOCX)
