# Supplementary figures and images for: 1H NMR Metabonomics Indicates Continued Metabolic Changes and Sexual Dimorphism Post-Parasite Clearance in Self-Limiting Murine Malaria Model
Source: PLoS One. 2013 Jun 24;8(6):e66954. doi: 10.1371/journal.pone.0066954 (PMC3691208; doi:10.1371/journal.pone.0066954)

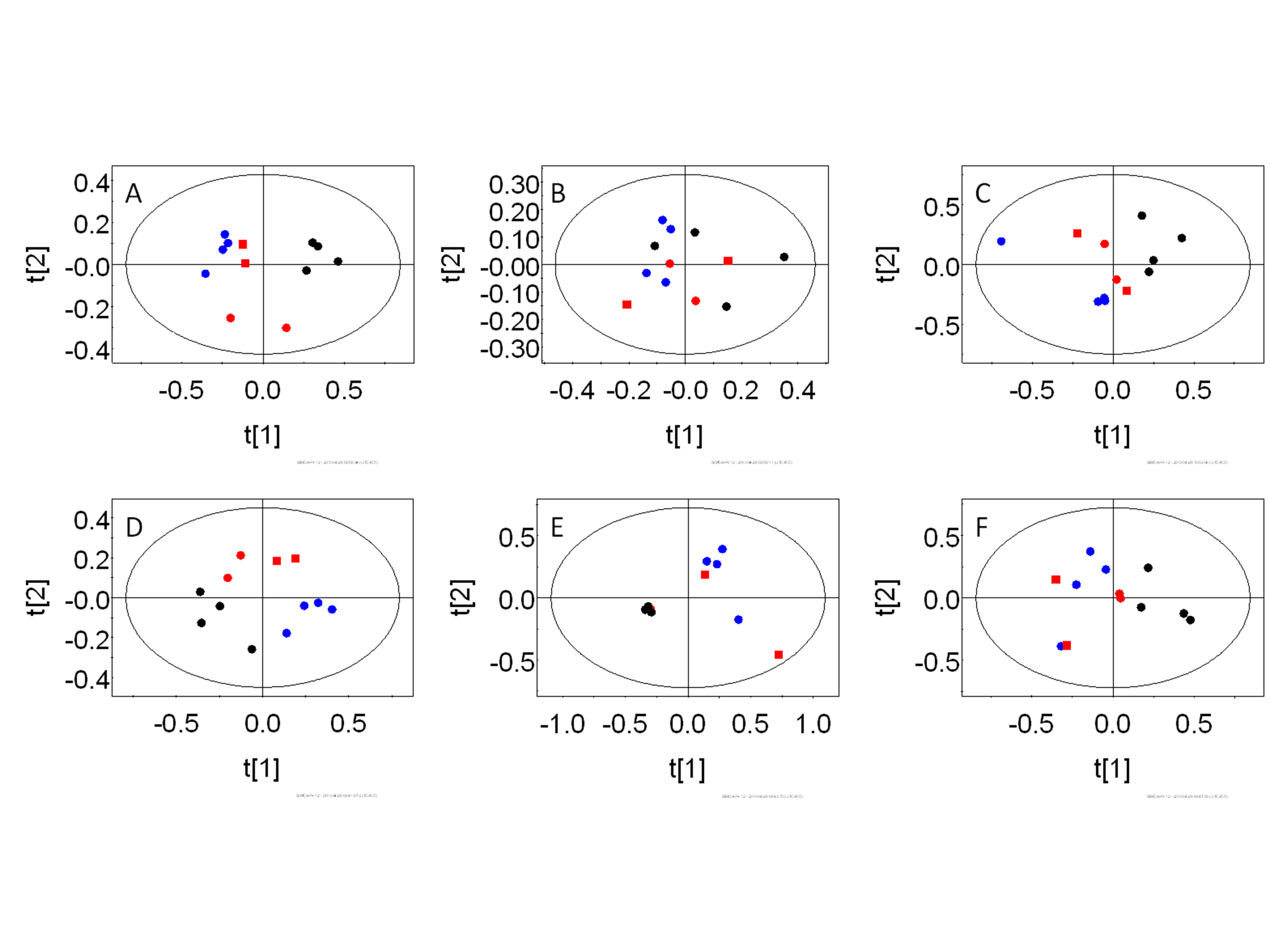

Supplement: Figure S1 — The PCA scores plots constructed from the NMR profiles of hydrophilic fractions of water/methanol/chloroform extracts. Female (A–C) and male (D–F) liver (A/D), brain (B/E) and blood serum (C/F) are shown. All the plots are constructed from the 1st and 2nd PCs of the models. In each plot, red dots- uninfected control animals, black dots- peak infection stage animals and blue dots- three weeks post parasite clearance animals. The total explained variances by the 1st and 2nd PCs in each model are as follows- A- 0.72, B- 0.58, C- 0.59, D- 0.68, E- 0.83 and F- 0.57. (TIF) [file pone.0066954.s001.tif]

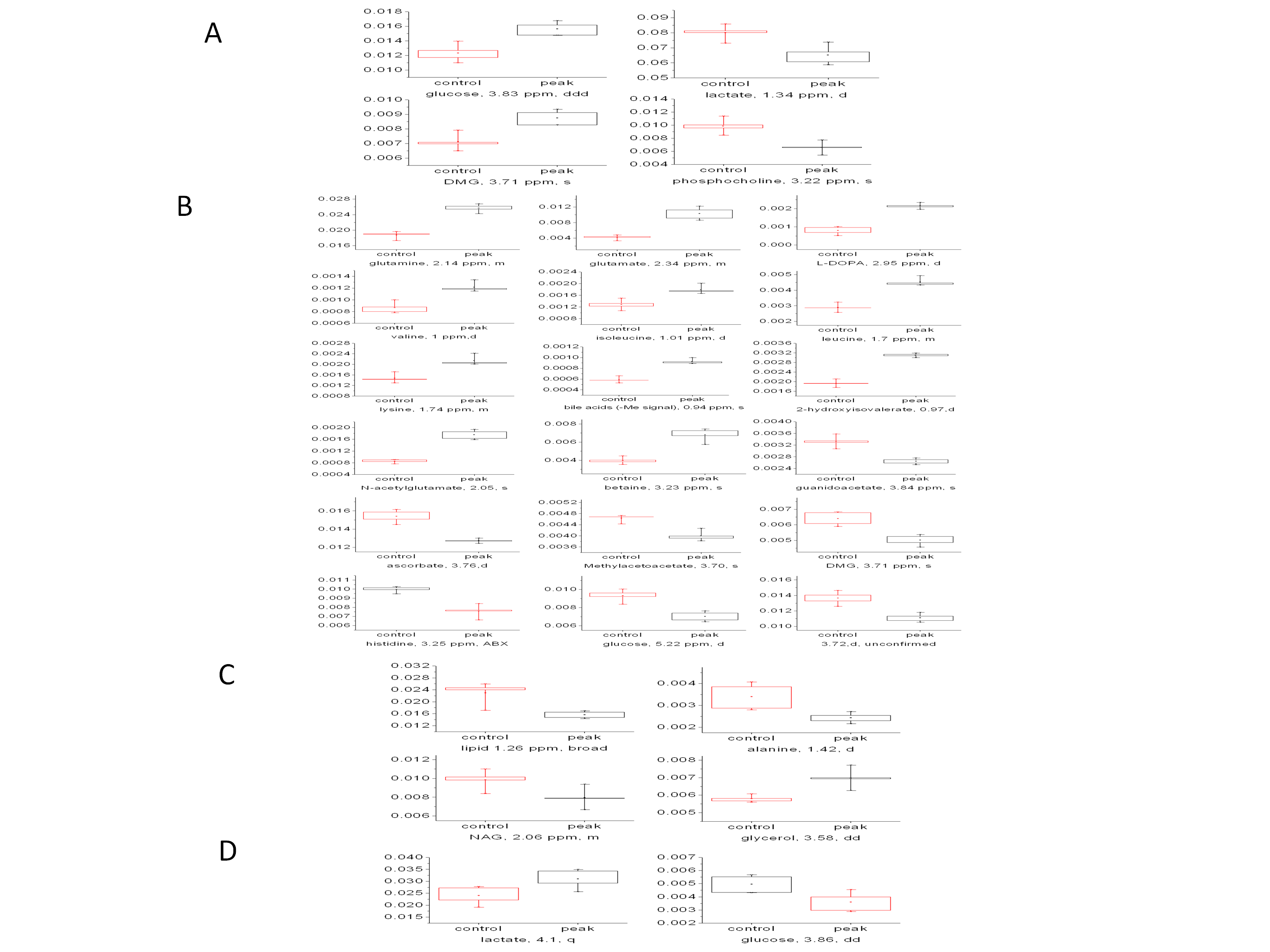

Supplement: Figure S2 — Relative changes of levels of the significantly perturbed metabolites during the peak infection stage compared to the uninfected controls illustrated by boxplots. A- male liver, B- female liver, C-male blood serum, D- female blood serum. The plots represents the comparison the NMR peak intensity normalized to total spectrum intensity (Y- axis) of the relevant metabolite peak at control (black) and peak infection stage (red) (X- axis). (TIF) [file pone.0066954.s002.tif]

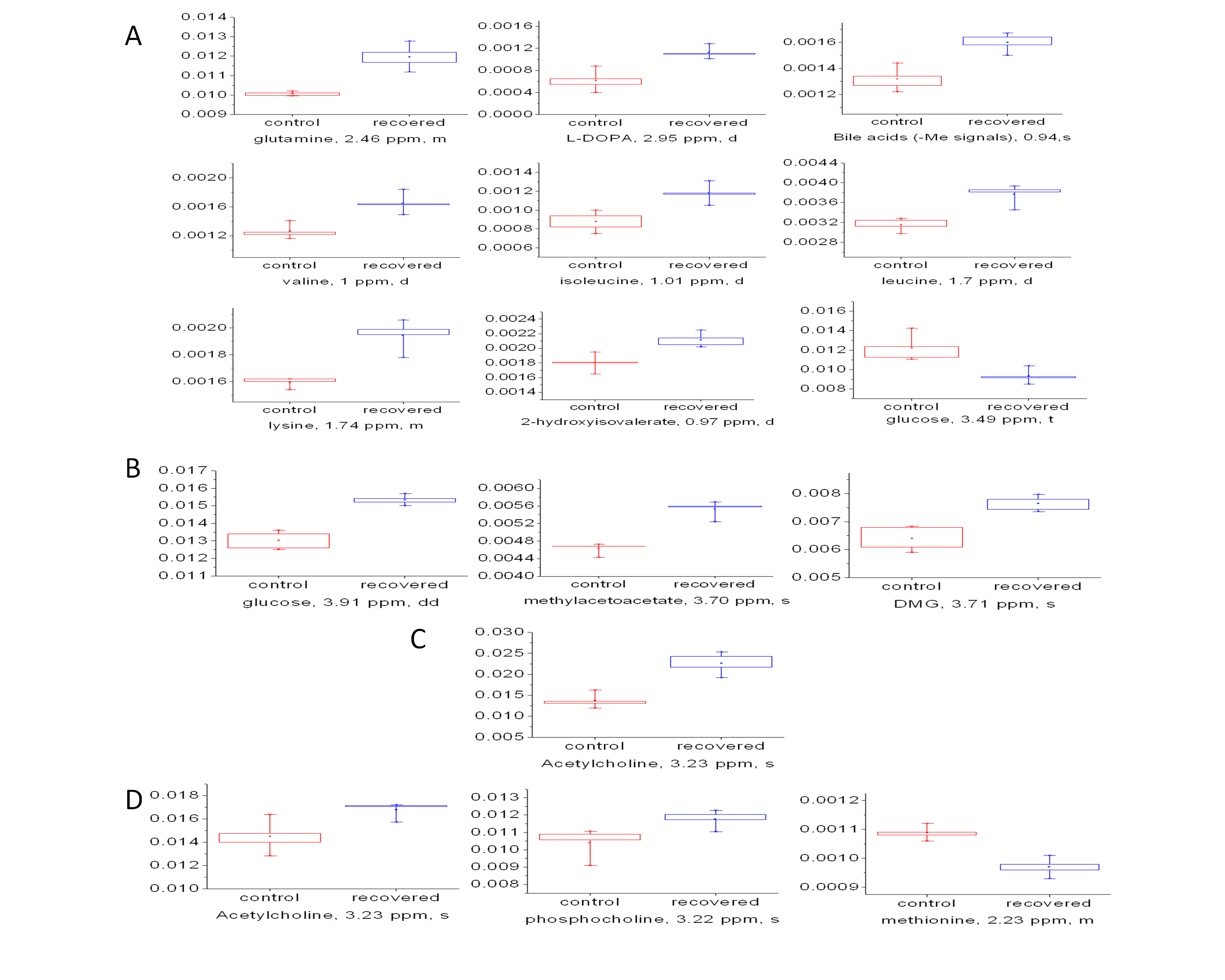

Supplement: Figure S3 — Relative changes of levels of the significantly perturbed metabolites during the three weeks post-parasite clearance stage compared to the uninfected controls illustrated by boxplots. A- male liver, B- female liver, C- male brain, D- female brain. The plots represents the comparison of the NMR peak intensity normalized to total spectrum intensity (Y- axis) of the relevant metabolite peak at control (black) and three weeks post-parasite clearance stage (red) (X- axis). (TIF) [file pone.0066954.s003.tif]
