# Supplementary material for: 1H NMR Metabonomics Indicates Continued Metabolic Changes and Sexual Dimorphism Post-Parasite Clearance in Self-Limiting Murine Malaria Model
Source: PLoS One. 2013 Jun 24;8(6):e66954. doi: 10.1371/journal.pone.0066954 (PMC3691208; doi:10.1371/journal.pone.0066954)
Supplement: Table S1 — CheBI IDs of the relevant metabolites reported. (DOCX) [file pone.0066954.s004.docx]

| Metabolite | | CheBI ID |
| --- | --- | --- |
| Glucose |  | 17634 |
| DMG |  | 17724 |
| lactate |  | 28358 |
| phosphocholine | | 18132 |
| glutamine | | 18050 |
| L-DOPA |  | 15765 |
| taurocholic acid | | 28865 |
| valine |  | 16414 |
| isoleucine | | 17191 |
| leucine |  | 15603 |
| lysine |  | 18019 |
| 2-hydroxyisovaleric acid | | 60645 |
| glutamate | | 16015 |
| N-acetylglutamate | | 17533 |
| betaine |  | 15870 |
| guanidoacetate | | 16344 |
| ascorbate | | 29073 |
| methylacetoacetate | |  |
| histidine |  | 15971 |
| acetylcholine | | 15355 |
| oxaloacetate | | 16452 |
| alanine |  | 16977 |
| methionine | | 16811 |
| glycerol |  | 17754 |
| lipoprotein | | 6495 |
| creatine |  | 16919 |
